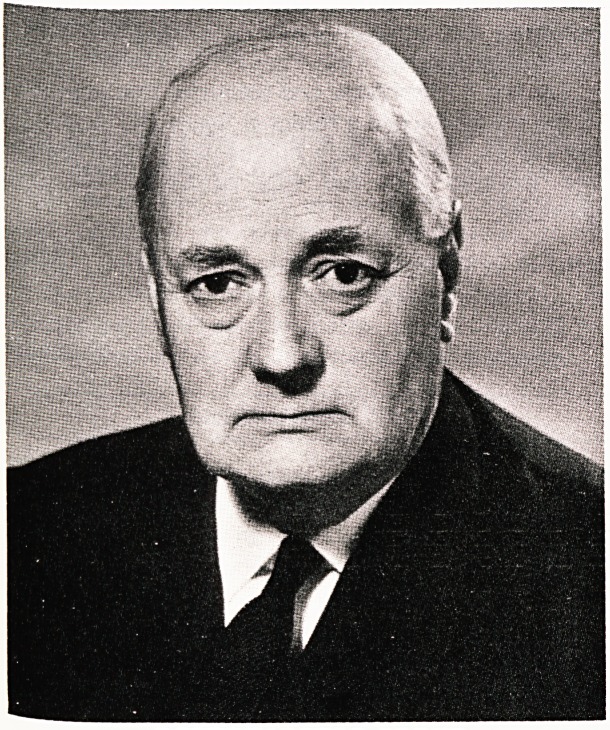# Robert V. Cooke

**Published:** 1978

**Authors:** 


### Obituary

**ROBERT VICTOR COOKE**
**M.D., Ch.M., F.R.C.S.**

Mr. Robert Cooke formerly Senior Surgeon to the United Bristol Hospitals died on 17th January 1978 at the age of 75.

He was born at Berkeley Gloucestershire and educated at the Grammar School at Lydney on the opposite side of the Severn Estuary.

He entered the University of Bristol to study Chemistry but soon moved to the Faculty of Medicine and qualified in 1926. After house surgeon appointments in Bristol he became House Surgeon to Cecil Joll at the Royal Free Hospital and it was with this great pioneer in thyroid surgery that his special interest and skill in this branch of surgery was formed. It was here also that he met Elizabeth Cowie, M.D., M.R.C.P., the Resident Medical Officer who in 1929 became his wife. In this year also he took the F.R.C.S. and in the following year the Ch.M. From the Royal Free he moved to Cardiff as Lecturer in Surgery and assistant to Professor Sheen.

In 1933 he was appointed Honorary Surgeon to the Bristol General and the Bristol Children's Hospitals and later to the Homeopathic Hospital. He also became Consultant Surgeon to Tetbury Hospital and for a time also at Southmead Hospital.

After the war he became a Member of the Court of Examiners of the Royal College of Surgeons and in 1956 was elected to the Council of the College becoming Senior Vice President in 1972.

Among his many honours within the College he was a Hunterian Professor and also gave a Hunterian Oration. In 1963 he was awarded the Cecil Joll Prize for Meritorious Service in general surgery and in 1970 he was invited to give the Bradshaw Lecture. He was active as a member of the R.S.M. and became President of the section of Surgery and later of Proctoscopy.

In 1967/68 he was President of the B.M.A.

The City of Bristol honoured him by making him Sheriff and the University with an honorary degree of Doctor of Medicine.

Though he was active in so many fields and achieved the Presidency of the Bristish Medical Association of surgical sections of the R.S.M. and the Vice Presidency of the R.C.S., he was first and foremost and always remained a working surgeon.

His capacity for work was remarkable, perhaps because his skill as an operator made the exercise of it a relaxation rather than a labour. His great technical skill was always kept in bounds by a judgement which never let him exceed the limits of the safe and the feasible. He was indeed a very safe surgeon — innately conservative and for this reason not an innovator.

He cared intensely about his patients and his anxieties about them were felt all down the line.

He was a memorable undergraduate teacher and conscientious in his duty to his students as well as a regular attender at clinical meetings where his contributions were always illuminating and drawn from his great experience and remarkable memory.

In his youth he had been a keen sportsman captaining the University Hockey team and playing for his county. Later golf was his main athletic outlet and for most of his life he managed to spend Thursday afternoon on Failand Golf Course.

He was a connoisseur of beautiful things and chose the title of 'Craftsmanship' for his Presidential address to the section of surgery of the R.S.M.

He was a lifelong collector of antique furniture and works of art and when he acquired Tudor Athelhampton they found their perfect home. To the many who have visited Athelhampton, which is now open to the Public, this is one of England's most beautiful homes. It is now occupied by his son, Mr. Robert Cooke, MP. To have rescued it from decay, restored it and filled it with beautiful things is an achievement of which he was justly proud.

The tragic death of his wife Elizabeth in 1964 was very hard for him to bear. They had two sons, the older Robert is MP for Bristol West. The younger, Christopher is a schoolmaster and teaches at Ravenscroft School near Bath. In 1970 he was married again to Dr. Mavis Coutts, a general practitioner and whose father was also in practice in Bristol. She had formerly worked for him for some years as house surgeon and clinical assistant at the Homeopathic Hospital. She restored to him the happiness and companionship of a home life during his last eight years.

He was a formidable and colourful character, and in the medical scene a National figure. To those who worked for him and with him he was a warm hearted and sensitive man and they remember him with gratitude.

## Figures and Tables

**Figure f1:**